# Reading outcomes in children with developmental language disorder: A person-centered approach

**DOI:** 10.1177/2396941520979857

**Published:** 2020-12-22

**Authors:** Marja C Erisman, Elma Blom

**Affiliations:** Faculty of Social and Behavioral Sciences, Utrecht University, Utrecht, the Netherlands

**Keywords:** Developmental Language Disorder, reading difficulties, phonology, executive functioning, person-centered approach

## Abstract

**Background and aims:**

Many children with Developmental Language Disorder (DLD) develop reading difficulties. The purpose of this study is to better understand variation in the reading outcomes of children with DLD using a person-centered approach.

**Method:**

87 monolingual Dutch children diagnosed with DLD performed at ages 5 or 6 years nine tests of nonverbal IQ, oral language proficiency, phonological memory (PM) and executive functioning (EF). Two years later, the same children were tested on single (non-)word reading. Latent profile analyses were conducted to identify profiles based on oral language proficiency, phonological memory and executive functioning at age 5–6 years, which, in turn, were related to nonverbal IQ and to single-word reading two years later.

**Results:**

Four profiles were identified and labelled relative to their position within the DLD-sample: 1. Weak performance overall, 2. Strong EF-average language and PM, 3. Mild working memory (WM) deficiencies-average language and PM, 4. Strong development overall. Profiles 1 and 3 had below average nonverbal IQ scores and were associated with low word reading outcomes two years later.

**Conclusions:**

Within the group of children with DLD, children with relatively weak oral language, phonological memory and executive functioning, or children with working memory deficiencies are most at risk for developing reading difficulties. The findings support a multiple risk framework and confirm that a person-centered approach is promising in predicting reading outcomes in DLD.

**Implications:** Research into individual differences in DLD is dominated by variable-centered approaches. This study illustrates how a person-centered approach, which views variables as properties of individuals, captures variation in the DLD-population. Using this bottom-up approach, the study highlights how an individual’s strengths and weaknesses across different developmental domains can be combined into profiles that relate to later reading outcomes. As such, it can provide an example for future DLD research.

Learning language and becoming literate is essential for a child’s wellbeing and success later in life. Many children with a Developmental Language Disorder (DLD) struggle with both language and literacy. DLD is a clinical condition that severely impairs oral language learning, despite adequate language input, normal hearing, and nonverbal intelligence (Leonard, 2014). It occurs frequently and affects about 7% of the population with a higher prevalence among boys than girls (Law et al., 2000; Tomblin et al., 1997). Severity of the impairment and affected domains show high variability (Bishop, 2017; Lancaster & Camarata, 2018). On top of their oral language problems, many children with DLD have difficulties with written language, and develop reading problems (Bishop et al., 2009; De Bree et al., 2012). It is largely unknown which children with DLD develop reading difficulties (Bishop, 2014).

Previous research about sources of individual differences in the reading skills of children with DLD typically uses a variable-centered approach (e.g., De Bree et al., 2012; Ramus et al., 2013; Rispens & Baker, 2012). Variable-centered approaches investigate relationships between predictors and outcomes, assuming that the population is *homogeneous.* Although these approaches are appropriate for examining the relative importance of predictors in explaining variation in outcome variables (Laursen & Hoff, 2006), they can lead to mixed and inconclusive results across studies examining a population that is *heterogeneous,* such as children with DLD. For such populations, a person-centered approach may be more adequate. Person-centered approaches focus on identifying distinct profiles of individuals based on response patterns of individual characteristics, in order to create groups with individuals that are more similar within groups than between groups (Jung & Wickrama, 2008). In the current study we applied a person-centered approach to better understand the reading outcomes of children with DLD.

## Learning to read: A multi-component development

**Figure 1.**
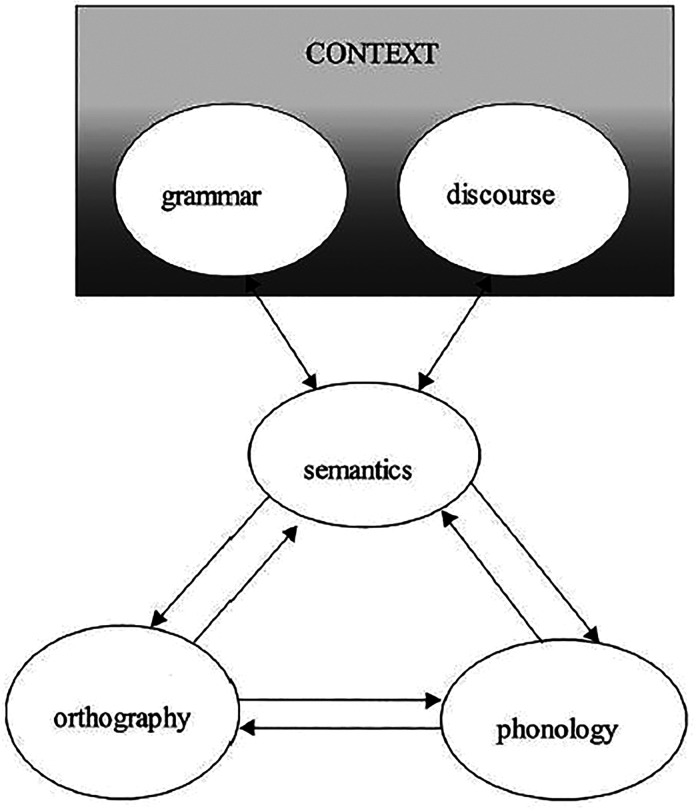
Learning to read according to the extended triangle model. Adapted from “Developmental Dyslexia and Specific Language Impairment: Same or Different?” by D. V. Bishop and M. J. Snowling, 2004, *Psychological Bulletin*, 130, p. 876. Copyright 2004 by the American Psychological Association.

Learning to read is a dynamic and interactive process and deficits in reading can be multi-causal (Bishop & Snowling, 2004). Several theories of literacy explain how children develop reading skills. A well-known example is the triangle model (Seidenberg & McClelland, 1989; Seidenberg, 2005), which suggests that two interactive pathways are developed; a phonological pathway maps orthography (printed words) to phonology (spoken words) and a semantic pathway maps orthography onto phonology via semantics (word meaning). According to Ehri (2014), the formed connections between written units and spoken units, which are maintained in memory along with word meanings, allow the reader to recognize words by sight. Bishop and Snowling (2004) extended the triangle model by emphasizing the relevance of grammatical knowledge and discourse skills (Figure 1). Grammatical knowledge facilitates single-word reading (Verhoeven et al., 2003) as well as text reading, while discourse skills are especially important for text reading. Children can develop reading problems due to deficits within each of the relevant domains identified in the triangle model. Unlike other theories of literacy, this model acknowledges that difficulties with mappings *between* the domains contribute to children’s reading problems and that the development of mappings may be constrained by cognitive limitations, such as being unable to retain and update information in working memory and scarce cognitive learning resources (Bishop & Snowling, 2004, p. 872).

## Reading in children with DLD

A large percentage of children with DLD have difficulties with reading and meet the criteria for dyslexia (Bishop, 2014). Dyslexia is diagnosed when children experience difficulties with written language (i.e., fluent word recognition, decoding, and spelling abilities), despite adequate input and abilities within the normal range on other academic domains (American Psychiatric Association, 2013). A study by McArthur et al. (2000) showed that 51% of children with DLD met criteria of dyslexia. Yet, there are also cases of pure DLD and dyslexia (Ramus et al., 2013), and both groups have different symptoms and underlying problems (Catts et al., 2005; De Bree et al., 2010). Therefore, DLD and dyslexia are best viewed as two distinct heterogeneous disorders that commonly co-occur (see Adlof & Hogan (2018) for a review).

Variable-centered studies on DLD and dyslexia identified phonological processing and oral language proficiencies as significant predictors of reading outcomes in children with DLD (e.g., Bishop et al., 2009; Ramus et al., 2013). Phonological processing concerns the use of sounds to process written and spoken language (Wagner & Torgesen, 1987). It includes a variety of skills, such as the perception of phonemes, encoding and momentary storage of phonological information, retrieval of this information and articulation (De Bree et al., 2010; Rispens & Baker, 2012). Deficits in phonological processing lead to poorer and slower generalization of letter-sound correspondences (Seidenberg, 2005). Several studies indeed found that children with DLD+dyslexia experience substantial difficulties in phonological processing (e.g., Ramus et al., 2013; Rispens & Baker, 2012). The relationship between phonological processing and reading weaknesses in DLD seems, however, more prominent in 4-year old children than in 9-year-olds (Bishop et al., 2009). Moreover, De Bree and colleagues (2010) found that all 4-year-olds with DLD had poor phonological processing skills, but phonological processing at age 4 years did not predict children’s reading outcomes four years later. These findings stress the need for further longitudinal research into the phonological predictors of reading ability in children with DLD. As part of the current research, we focused on phonological memory, which plays a significant role in phonological processing (Rispens & Baker, 2012).

The extended triangle model (Bishop & Snowling, 2004) posits that multiple abilities play a role in reading, including language abilities beyond the level of phonology. The few studies that examined oral language proficiency present discrepant results. The study by Ramus et al. (2013) showed that children with DLD+dyslexia were more severely impaired on morphology, syntax, and vocabulary than children with DLD-only. Bishop and colleagues (2009) found that children with DLD-only and DLD+dyslexia did not differ significantly on vocabulary, understanding directions and narrative skills, but the children with DLD+dyslexia performed lower on repeating sentences, which could be taken as an indication of poor syntactic skills (Polišenská et al., 2015). In contrast, Catts et al. (2005) did not observe any differences in vocabulary, morphology, and syntax (including sentence repetition) between children with DLD+dyslexia and children with DLD-only. These inconsistent findings regarding the role of oral language proficiency necessitate further research into the relationships between different measures of oral language proficiency and reading in children with DLD.

A third domain relevant to the reading outcomes of children with DLD is domain-general cognition, an umbrella for subdomains such as the executive functions and nonverbal intelligence. Executive functions are cognitive functions used for goal-oriented and flexible behavior (e.g., working memory, interference control (or inhibition), and attentional shifting) (Miyake et al., 2000). Children with DLD tend to perform low on tasks testing executive functioning (Pauls & Archibald, 2016), and this also holds for the DLD sample investigated for the current research (Blom & Boerma, 2020). Moreover, research suggest that impairments in executive functioning are related to reading difficulties (Booth et al., 2010; Brosnan et al., 2002), especially deficits in working memory and interference control (Brosnan et al., 2002). Adopting Baddeley's (1986) working memory model, Schuchardt et al. (2013) found that two working memory components, the phonological loop and aspects of the central executive, were more severely impaired in children with DLD+dyslexia than in a dyslexia-only group. The central executive overlaps with the executive functions (McCabe et al., 2010), suggesting that executive function limitations could contribute to the reading difficulties of children with DLD. A recent study by Gray et al. (2019) confirmed that children with DLD+dyslexia are more likely to have low working memory than children with DLD-only or dyslexia-only.

Comparing children with dyslexia-only, DLD-only, DLD+dyslexia and TD, Gray et al. (2019) found that particularly many children in the DLD+dyslexia group with lower nonverbal IQ scores were in the lowest performing working memory profile. Nonverbal intelligence refers to higher-order cognitive skills that enable one to make sense of the world without necessarily using words (e.g., abstract reasoning, problem solving, decision making skills). Working memory, and, more in general, executive functioning, is closely linked to nonverbal intelligence though not identical (Conway et al., 2003; Engelhardt et al., 2016): Children may exhibit executive functioning deficits independent of their nonverbal IQ scores (Henry et al., 2012; Kuusisto et al., 2017). Nonverbal intelligence is also related other predictors of reading in children with DLD. There is, for example, evidence suggesting that children with DLD with low IQ scores (i.e., -1 *SD* and -2 *SD*, corresponding to IQ scores between 71-84) tend to score lower on oral language measures than children with an IQ score of 85 or above (-1 *SD* or better) (Tomblin & Nippold, 2014; but see Norbury et al., 2016). In regard to reading, there is some evidence that nonverbal intelligence is significantly related to measures of single word reading in children with DLD (Botting et al., 2006).

In sum, there is theoretical and empirical support for the hypothesis that phonological, oral language, and executive function abilities predict the reading outcomes of children with DLD. Research suggests furthermore that predictors of reading outcomes of children with DLD across different domains are related to nonverbal intelligence. How these different abilities are related within the individual child, whether they are clustered into profiles and how these profiles are related to nonverbal intelligence and reading in DLD is unknown. To elucidate these issues, a person-centered approach may be promising.

## A person-centered approach

Most research at the intersection of DLD and reading difficulties uses a variable-centered approach, for example by conducting regression analyses (e.g., Catts et al., 2005; De Bree et al., 2012). Variable-centred approaches are the first step in identifying important predictors of an outcome. The outcomes of these approaches often lack clinical relevance, however, as reliable conclusions cannot be drawn. As Bishop (2017) states:

It is frustrating that even when we have evidence from longitudinal studies, the clinical application of the findings is often limited because of an emphasis on demonstrating that a predictor is statistically significant, rather than on its effectiveness in predicting individual outcomes. (p. 676)

Person-centered approaches such as latent profile analysis (LPA), can fulfill the need for studies that investigate individual differences within a heterogeneous population, such as children with DLD. To understand relations with specific problems, including reading problems, it may be necessary to identify homogeneous classes within the heterogeneous DLD population (Bishop, 2014). This can be achieved with LPA.

Lancaster and Camarata (2018) argued that the variability in severity and presentation of symptoms within the DLD population *cannot* be explained by different meaningful profiles or groups of individuals. In their view, a continuum or spectrum approach, as done in Austisme Spectrum Disorder (see DSM-IV and DSM-V; American Psychiatric Association, 2000, 2013), is preferred over an approach that identifies subtypes and leads to higher accuracy of the diagnoses (e.g., Frazier et al., 2012) and better “levels of support for all individuals on the spectrum” (Lai et al., 2013, p. 2). Conceptualizing DLD as a spectrum disorder has benefits for clinical settings, but the spectrum approach yields difficulties within research practices. Treating a heterogeneous condition as a spectrum disorder may lead to too much noise in research data, which renders useless results and hinders the development of effective interventions (Lai et al., 2013; Wiggins et al., 2017). It is thus important to distinguish between forming *clinical subtypes* to reach diagnostic consensus and forming *subgroups* using bottom-up data generated techniques to understand the various etiologies of the disorder (Wiggins et al., 2017). Although it is likely that a spectrum approach that focuses on individual traits and severity will improve accuracy of the DLD-diagnosis (Lancaster & Camarata, 2018), formulating differences and similarities between subgroups is necessary to understand the complex nature of DLD.

For these reasons, we used in this study a person-centered approach to explore which children with DLD are susceptible to developing reading problems. In DLD research on subtypes, nonverbal intelligence plays a prominent role (Rice, 2016). It is debated whether it makes sense to use cut-off scores for nonverbal intelligence and distinguish between children with DLD who have average nonverbal intelligence and low-average nonverbal intelligence, or between DLD and a more general intellectual disability (Bishop, 2017; Lancaster & Camarata, 2018). As poor nonverbal intelligence can affect learning in general (Neisser et al., 1996), taking nonverbal intelligence into account is valuable when investigating possible predictors of poor oral and written language learning (Bishop, 2014). Therefore, we wanted to know whether and how the profiles detected using LPA are related to nonverbal intelligence.

## This study

The goal of this study was to answer the following overarching research question: Which children with DLD develop reading problems? To answer this question, this study explored, first, whether latent profiles could be identified in a DLD sample consisting of Dutch-speaking monolinguals aged 5 or 6 years old, based on multiple measures of oral language, phonological memory and executive functions. Second, it was examined how these latent profiles were related to nonverbal IQ scores. Third, we explored whether these profiles predicted reading outcomes (i.e., single (non-)word reading) two years later. As the DLD population is known for its heterogeneity (Leonard, 2014; Lancaster & Camarata, 2018), we hypothesized a) that different profiles within the DLD-sample can be identified, which are distinguished by varying abilities in phonological memory, oral language proficiency and executive functioning, b) that these profiles are related to nonverbal intelligence, and c) that they differ with regard to reading outcomes, as the aforementioned skills are susceptible to developing reading difficulties (Bishop et al., 2009; Booth et al., 2010; Ramus et al., 2013).

To answer the research question, we analyzed longitudinal data from a group of children with DLD between ages 5 and 8 years, thereby building on two previous studies about the same DLD sample as investigated for the purpose of the current study. One study demonstrated that executive functioning predicted receptive vocabulary in children with DLD (Blom & Boerma, 2019). In the other study, it was observed that children with DLD had lower outcomes on non-verbal executive functioning (Blom & Boerma, 2020). This effect was most prominent in children with severe and persistent DLD. For the current study, it is relevant that both studies suggest relationships between oral language and executive functioning, which can be direct or indirect. It is unknown, however, whether oral language, phonological memory, and executive function abilities cluster into profiles, how these profiles are related to nonverbal intelligence and if these profiles are related to later reading outcomes. The current study fills these empirical gaps and contributes to our understanding of the mechanisms that underlie reading skills in children with DLD.

## Method

### Participants

For the purpose of the current study, longitudinal data from monolingual Dutch children were analyzed. Data collection took place between 2014 and 2016 and comprised three waves of testing, with one year between each wave of data collection. For the purpose of the current study, we selected the data that were collected at the beginning of the study (*N* = 87) and two years later at the end of the study (*N* = 86). We will refer to this as time 1 and time 2. The reason for the selection is that we wanted to investigate how early profiles relate to later reading outcomes and provide insight into prediction or heterotypic stability (Bornstein et al., 2017).

Children with DLD were selected based on their diagnosis and recruited via two national healthcare institutions: Royal Auris Group and Royal Dutch Kentalis. Before the start of the project, participants had been officially diagnosed by licensed clinicians according to standardized criteria. Standardized criteria included an obtained score of minimally 2 standard deviations (*SD*) below the population mean on their overall score on a language assessment test battery, or an obtained score of minimally 1.5 *SD* below the population mean on two out of four subscales of this language assessment test battery (Stichting Simea, 2014). In the Netherlands, the *Schlichting Test for Language Production and Comprehension* (Schlichting et al., 1995) and the Dutch version of the *Clinical Evaluation of Language Fundamentals* (CELF-4-NL; Kort et al., 2008) are the most commonly used standardized language test batteries.

At time 1, all 87 children in the DLD-group met the specified standardized criteria for DLD. At time 2, 26 children no longer met these criteria. These children were not excluded. Language problems are known for their long-term persistence (Johnson et al., 1999), and failing to meet the arbitrary cut-offs linked to a DLD diagnose does not imply that the language problems are resolved. The latter was confirmed in our previous research, in which we demonstrated that the children in the sample who were not diagnosed with DLD anymore did perform lower than typically-developing controls on several language measures (Blom & Boerma, 2020). Children with hearing loss, neurological impairments (e.g., epilepsy), severe articulatory difficulties and comorbid disorders (e.g., ASD) were excluded from this study.

Children (74.7% male) were aged 5 or 6 at time 1 (*M*_months_ = 71.53, *SD*_months_ = 6.58), with two exceptions. One child was 4 years old and one child was 7 years old at time 1. At time 2, children were 7 or 8 years old (*M*_months_ = 94.52, *SD*_months_ = 6.59). However, two children were aged 6 years old and one child was 9 years old. Out of the 87 participants with DLD, 61 children attended special education at time 1. The other children attended regular education with ambulatory care. Between time 1 and 2, 16 children transferred to regular education, of whom 12 received ambulatory care. Furthermore, 3 children transferred from regular education to special education. One child did not continue to participate between time 1 and 2. The children in the sample varied with respect to social economic status (SES) indexed by parental education. The mean parental education level on a nine-point scale, ranging from *no education* to *university degree*, was 5.47 (*SD* = 1.79; average of both parents), which equals vocational education.

### Procedure and measures

The Standing Ethical Assessment Committee of the Faculty of Social and Behavioral Sciences at Utrecht University approved this project. Parents of participants signed informed consent forms. Each wave, children were asked to perform several tasks, as discussed below. Nonverbal IQ was only assessed at the first wave (time 1 in the current study) and reading ability was only assessed at the third wave of data collection (time 2 in the current study). Measurements of oral language proficiency, phonological memory and executive functioning were assessed at each wave, but the current study only used the measurements of time 1. Testing took place in two sessions in a quiet room at the child’s school, each session lasting approximately one hour. Children were individually tested by a trained research assistant who is a native speaker of Dutch.

#### Reading ability

Reading performance was measured using the *Eén Minuut Test* (One Minute Test [EMT]; Brus & Voeten, 1979) and the *Klepel* (Van den Bos et al., 1994). The child had to read unrelated single words (EMT) and single non-words (Klepel) as quickly and accurately as possible in one and two minutes respectively. In both tasks, word length gradually increased from one to four syllables. The raw scores reflect the number of words read correctly within time limits (ranging from 0-116 per task) and were transformed into age-normed scaled scores (*M* = 10, *SD* = 3). Scores below 5 reflect a very weak score, between 5-7 a weak score, between 8-12 an average score, between 13-15 an above average score and above 15 a high score. Internal consistency has been found to be excellent, specifically .92 for Klepel and .90 for EMT (Evers et al., 2009–2012).

#### Oral language proficiency.

Oral language proficiency was measured using three standardized language tests that evaluated children’s receptive vocabulary, grammatical morphology and sentence repetition. Receptive vocabulary was measured with the Dutch version of the *Peabody Picture Vocabulary Test* (PPVT-III-NL; Schlichting, 2005). The PPVT-III-NL is a widely used standardized test in which children choose the correct picture out of four, matching a verbally presented target word. The task includes 17 sets, each containing twelve items gradually increasing in difficulty. The starting set was determined based on the child’s age and the task was terminated when an incorrect picture was chosen nine or more times within one set. Raw scores reflect the number of correctly picked pictures. The PPVT-III-NL has been found to be valid and reliable, with a test–retest reliability of .94 (Schlichting, 2005).

Grammatical morphology was measured with the subtest Word Formation of the *Taaltoets Alle Kinderen* (Dutch Language Test for All Children [TAK]; Verhoeven & Vermeer, 2006). Children were presented with an image and asked to complete a sentence, eliciting the plural of the noun or the past participles of a verb. The task consisted of 24 items, with 12 items targeting plurals and 12 items targeting past participles. Both regularly and irregularly inflected nouns and verbs were included. Raw scores reflect the number of correct answers (24 maximum). Internal consistency has been found to be good, ranging from .89 to .91 (Verhoeven & Vermeer, 2006).

Sentence repetition was measured with the subtest Sentence Formation of the TAK (Verhoeven & Vermeer, 2006). Sentence repetition tasks measure several skills related to children’s sentence level abilities (lexicon, syntax, verbal short-term memory), but primarily taps into a child’s syntactic skills (Polišenská et al., 2015). Children had to repeat 20 sentences, which varied from nine to 15 words in length. Each sentence was scored on the accurate repetition of a function word and a sentence pattern. Independent scoring of function words and sentence patterns led to a maximum score of 40. Internal consistency has been found to be excellent, ranging from .91 to .96 (Verhoeven & Vermeer, 2006).

#### Phonological memory

Phonological memory was measured using the Digit Span Forward task based on the *Alloway Working Memory Assessment* (AWMA; Alloway et al., 2006). The Digit Span Forward is a measure of phonological short-term memory, which plays a significant role in phonological processing (Rispens & Baker, 2012). In this task, children were asked to repeat a sequence of digits in the same order as presented. Each block contained six trials and there was a maximum of seven blocks. The number of digits within one sequence increased over the blocks, starting with one digit in the first block. Each correct trial was awarded with one point, up to a maximum score of 42. If children correctly answered the first four trials of a block, they automatically continued with the next block and received the maximum of six points. The task ended after three incorrect responses within the same block. The AWMA has been found to be valid (Alloway et al., 2008) and reliable, with test–retest reliability of .84 for the Digit Span Forward (based on a sample of children aged 4.5–11.5 years; Alloway et al., 2006).

#### Executive functioning

The present study included measures of interference control, selective attention and verbal and visual-spatial working memory. Interference control was measured with a child-friendly version of the Flanker task (Eriksen & Eriksen, 1974), adapted by Engel de Abreu et al. (2012). The online task was completed on a computer screen on which five equally spaced yellow fish were presented on a horizontal row. Children indicated the direction of the central fish by quickly pressing the corresponding right or left response button which were placed on each side of the computer screen. Half of the trials consisted of the central fish pointing in the same direction as the other fish (congruent trials), and the other half consisted of the central fish pointing in the opposite direction (incongruent trials). At the beginning of each trial a 1,000 ms fixation cross was shown in the middle of the screen, after which the fish array was presented for 5,000 ms or until a response was made. Congruent and incongruent trials were randomized in two blocks of 20 trials. Before the start of the test, children completed eight practice trials. Reaction times (RTs) and accuracy were registered. The mean RTs were calculated including only correct responses, RTs above 200 ms and RTs below three standard deviations of children’s individual means. In the analyses, we used the Flanker (or congruency) effect outcomes, which are calculated by subtracting the mean RT of the congruent trials from the incongruent trials. A large Flanker effect indicates that interference caused by flanking fish that look in the opposite direction impacts a child’s performance strongly, suggesting limited abilities to control interference. As such, the size of the Flanker effect provides valuable information on the mechanisms involved in controlling interference (Hübner & Töbel, 2019). We did not include accuracy scores, because these were high (i.e., 84% correct). No psychometric information is available for this task.

Selective attention was measured with the visual Sky Search subtest from the *Test of Everyday Attention for Children* (Manly et al., 2001). Children were presented with 128 pairs of spaceships on a A3 sheet paper. Children were asked to encircle the identical pairs (20) as quickly as possible while ignoring the non-identical pairs of spaceships and say “stop” when they thought they finished. Subsequently, children were represented with a new A3 sheet containing only the 20 identical pairs. Children were asked to encircle these pairs as quickly as possible. To adjust for motor speed, children’s attention score was calculated by subtracting the mean time per target of the second condition from the mean time per target of the first condition. The test-retest reliability of the visual Sky Search ranges from .75 to .90 (Manly et al., 2001).

Verbal working memory was measured with the Digit Span Backward based on the AWMA (Alloway et al., 2006). Children were asked to verbally repeat sequences of digits in reversed order. Sequences gradually increased in length, up to seven digits maximum. Visual-spatial working memory was measured with the Dot Matrix Backward based on the AWMA (Alloway et al., 2006). Children were presented with a 4x4 matrix in which a red dot appeared on different locations in a sequence and were asked to remember this in reversed order. The number of dots within a sequence increased gradually over the blocks. The task consisted of six blocks maximum, each containing six trials. See phonological memory for the AWMA scoring procedure. Children could obtain a maximum score of 42 for verbal working memory and 36 for visual-spatial working memory. The test–retest reliability for the Digit Span Backward was .64 (Alloway et al., 2006), for the Dot Matrix Backwards it is unknown.

#### Nonverbal intelligence

Nonverbal intelligence was measured using the short version of the *Wechsler Nonverbal Scale of Ability* [WNV-NL], which consisted of two subtests (Wechsler & Naglieri, 2008). For the subtest Matrices, children were asked to select the missing figure of an incomplete figural matrix. For the subtest Recognition, children looked three seconds at a geometric design and were then asked to choose the matched stimulus. Raw scores reflect the number of correct items. Raw scores were converted to T-scores, which were then converted in a Full Scale Score, that had a mean of 100 and a SD of 15. Reliability of the Full Scale Score was .91 (Wechsler & Naglieri, 2008).

### Data analysis

To answer the research question, person-centered analyses were conducted in Mplus version 8.2, using maximum likelihood estimation (Muthén & Muthén, 1998–2017). Two outliers (*z*-score > 4.00) of selective attention were deleted and recoded as missing data. Missing data were handled with full information maximum likelihood estimation (FIML; Schafer & Graham, 2002). Missingness on each model indicator was low, with covariance coverage ranging from .98 to 1.00. A large number of random starts (i.e., 500 or 1000) was used to avoid that the likelihood function converged on local solutions.

To investigate latent profiles at time 1, a series of unconditional LPAs were specified in a step-by-step procedure, starting with a two-profile model. Each run, the number of profiles was increased by one, up to a six-profile model. The eight predictors of reading ability (i.e., vocabulary, morphology, sentence formation, phonological memory, interference control, selective attention, visual-spatial and verbal working memory) were used as profile indicators, which represented the three broader latent constructs (i.e., oral language proficiency, phonological memory, executive functioning). For reasons of parsimony, the assumption of local independence was implemented, which assumes that the correlation among the indicators within profiles is entirelty explained by the latent profile (Williams & Kibowski, 2016). Implementing this assumption ensured meaningful interpretations of the profiles and avoided unstable solutions.

Several model fit indices were used to compare the models and determine the best fitting LPA (Nylund et al., 2007). Firstly, the Bayesian Information Criterion (BIC) and sample size adjusted BIC (ABIC) were evaluated, with lower values representing good model fit. Secondly, the Lo-Mendell-Rubin test (LMR) and Bootstrapped Likelihood Ratio Test (BLRT) were used, which evaluated whether including an extra class significantly improved the model with *k*-classes (Nylund et al., 2007). Thirdly, the Bayes Factor (BF) was calculated, which assessed the probability that a model with *k*-classes is preferred over a model with *k +* 1 classes. Values between 1 and 3 indicated weak evidence for the *k-*classes model and values greater than 10 indicated strong evidence. Fourthly, the correct model probability (cmP) was computed, which provided the probability that a specific model was preferred compared to all models under consideration (Masyn, 2013). Lastly, entropy values were considered, which indicated the strength of the classification. Values above 0.80 indicated good classification (Nylund-Gibson & Choi, 2018). In addition to the statistical fit indices, the preferred model was evaluated on its interpretability and theoretical viability (Kam et al., 2013). This was examined with a profile plot for the model under consideration, which represented the profile-specific means of each indicator. To foster interpretability within the profile plot, the profile-specific means were rescaled to *z*-scores based on the sample mean and standard deviation (i.e., relative to their position within the overall DLD sample). Moreover, solutions with small numbers of children within profiles (i.e., less than 10) were not further considered (Nylund-Gibson & Choi, 2018).

Subsequently, interpretations of each profile in the chosen LPA were derived from the profile plot. The degree of general and indicator-specific profile separation was used as a method to validate the interpretation (Masyn, 2013) and evaluate the quality of the chosen latent profile solution (Geiser, 2013). General profile separation was evaluated by the average latent profile assignment probabilities. Values above .80 on the main diagonal of the matrix indicated that, on average, children were classified with high accuracy into their most likely latent profile (Geiser, 2013). The degree of profile separation between each profile on each indicator was measured by the distance between the profile specific means of each indicator *and* the variances of the distributions (Masyn, 2013). This was calculated by an adapted formula for Cohen’s *d* by Masyn (2013, p. 589). Values below 0.85 indicate a low degree of profile separation and values above 2.00 indicate a high degree of profile separation.

Differences in nonverbal IQ scores across latent profiles were examined using the Bolck-Croon-Hagenaars (BCH) approach (Bakk et al., 2013). This approach accounts for classification errors and avoids shifting between profiles by using a weighted multiple group analysis, in which the groups correspond to the posterior probabilities of latent profile membership (Asparouhov & Muthén, 2018). The BCH approach calculated profile-specific means of nonverbal IQ scores and conducted pairwise comparisons between profiles using the Wald χ^2^ test. To examine whether the profiles were related to later reading ability, differences in reading performances across latent profiles was also examined using the BCH-approach. The BCH approach calculated profile-specific means of both reading outcomes and conducted pairwise comparisons between profiles using the Wald χ^2^ test.

## Results

Descriptive statistics are followed by two subsections that describe the model building steps of the person-centered approach with first the results of the LPA, including a comparison between profiles and nonverbal IQ, and second the relation between the profiles and reading outcomes.

### Descriptive statistics

Table 1 summarized the means, range and number of children for vocabulary, morphology, sentence repetition, phonological memory, interference control, selective attention, verbal working memory, visual-spatial working memory, nonverbal intelligence, single word reading (time 2), and single non-word reading (time 2). The *SD*s of most measures are relatively large, confirming the heterogeneity of the disorder within DLD. See Table A1 in the appendices for correlations between all measures in Table 1.

**Table 1.** Descriptive statistics.

	*M (SD)*	Range	*N*
Vocabulary (time 1)	76.51 (11.73)	44–108	87
Morphology (time 1)	10.47 (4.03)	0–18	87
Sentence Repetition (time 1)	11.81 (8.01)	0–34	87
Phonological Processing (time 1)	14.90 (4.28)	1–24	87
Interference Control (time 1)	344.07 (478.41)	–898.64–1741.70	87
Selective Attention (time 1)	12.18 (8.13)	3.71–44.33	85
Verbal WM (time 1)	8.70 (3.50)	1–17	87
Visual-spatial WM (time 1)	10.36 (4.92)	2–24	87
Nonverbal IQ (time 1)	93.45 (18.11)	58–131	87
Single word reading (time 2)	6.87 (3.93)	1–16	86
Single non-word reading (time 2)	6.81 (3.69)	1–17	86

Note: WM = working memory.

### Profiles of children with DLD

Model fit indices for the LPAs are presented in Table 2. The BLRT was uninformative as its value was significant for each model analyzed. Although AIC, ABIC, BIC, cmP and BF supported the five- or six-profile model, these models were not further considered due to the small number of children within each profile. The VLMR-LRT indicated that a two-profile model was preferred. Profile plots for the two- to four-profile solutions were examined for their theoretical validity. The two- and three-profile models resulted all in rank-ordered solutions, with models representing lowest to highest scoring profiles on all measures. The four-profile solution, however, resulted in profiles that showed more differentiation on the measures between the profiles. Therefore, a four-profile solution was preferred. The entropy for this model was .90.

**Table 2.** Model fit indices for LPA.

Number of profiles	LL	AIC	BIC	ABIC	VLMR-LRT *p-*value	BLRT *p-*value	BF	cmP	Entropy	Minimal N
2	–1947.85	3945.70	4007.35	3928.47	**.007**	.000	<.001	<.001	.90	29
3	–1915.59	3899.19	3983.03	3875.75	.419	.000	2.38	.052	.91	10
4	–1898.16	3882.33	3988.36	3852.68	.293	.013	0.17	.004	.90	11
5	–1872.53	3849.06	**3977.29**	3813.21	.447	.000	**41.93**	**.922**	.93	5
6	–1856.17	**3834.34**	3984.76	**3792.28**	.169	.000	–	.022	.93	1

Note: Values presented in bold indicate the preferred model for that specific fit index.

**Figure 2.**
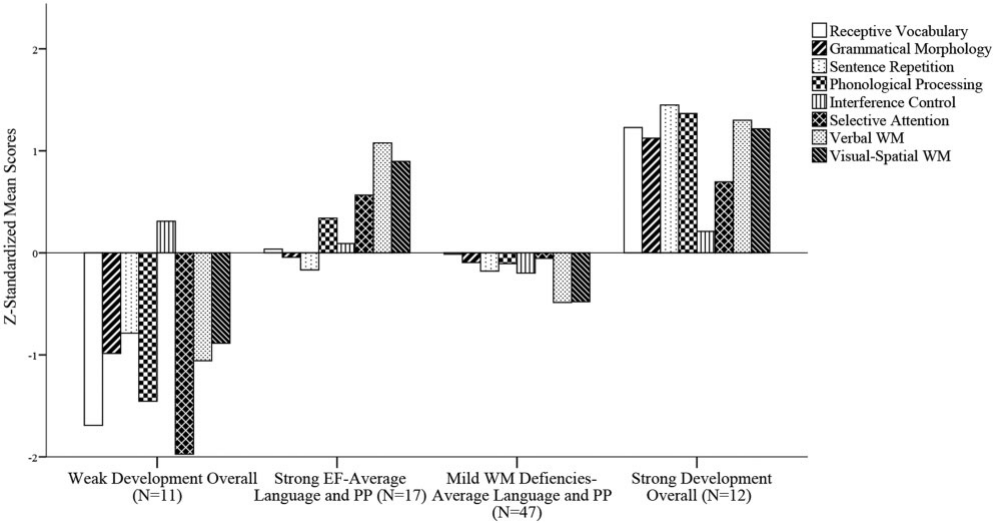
Profile Plot of the Four-Profile Unconditional LPA. Selective Attention and Interference Control were reversed for the sake of interpretability (i.e., a low score represents a weaker selective attention). Profiles were labelled relative to their position within the overall DLD sample. PP = Phonological Processing, EF = Executive Functioning, WM = Working Memory.

The profile plot of the four-profile solution is presented in Figure 2. Profile-specific means of each indicator can be found in Table 3. It is important to note that profiles were labelled relative to their position within the overall DLD sample. Specifically, labels were constructed in terms of *SD*s from their means (e.g., the label *weak* was given when *z*-transformed profile specific means were more than 1 *SD* below the sample mean, and *strong* was given in case specific means were more than 1 *SD* above the sample mean. Interference control did not differ substantially between the profiles (i.e., *z-*scores around zero in each profile) and was, therefore, not included in profile interpretation. However, interference control was retained within the analyses, as it is considered an important predictor for reading performances (e.g., Brosnan et al., 2002).

**Table 3.** Profile specific mean scores of children with DLD.

	Weak development overall (*N =* 11)		Strong EF-average language and PP (*N =* 17)		Mild WM deficiencies-average language and PP (*N =* 47)		Strong development overall (*N =* 12)	
Time 1	*M*	*SE*	*M*	*SE*	*M*	*SE*	*M*	*SE*
Receptive Vocabulary	60.31	3.17	77.16	2.16	76.33	1.79	90.41	3.30
Morphology	7.00	1.60	10.27	1.41	10.06	0.55	15.09	0.74
Sentence Repetition	6.35	1.14	10.40	1.81	10.33	1.23	23.40	3.26
Phonological Processing	8.27	1.96	16.34	1.14	14.40	0.51	20.41	0.79
Interference Control^a^	–.47	0.43	–.12	0.12	.22	0.21	–0.23	0.12
Selective Attention	26.83	5.18	8.09	1.32	12.48	1.01	6.55	1.34
Verbal WM	5.09	0.75	12.28	0.61	6.89	0.50	13.30	0.91
Visual-spatial WM	6.16	0.84	14.21	1.94	8.02	0.41	16.35	2.04

Note: Profiles were labelled relative to their position within the overall DLD sample. PP = Phonological Processing, EF = Executive Functioning, WM = Working Memory.

a Interference Control was z-standardized, because the variance of the raw scores was too large to be correctly handled in the analyses.

The first profile (12.64% of the sample) was labelled as *Weak Development Overall*. This profile was characterized by severe weaknesses in oral language abilities, executive functioning, and phonological memory. Children with this profile showed the largest deficiencies on selective attention and vocabulary. The second profile was labelled *Strong EF-Average Language and PM (*19.54% of the sample). Children with this profile had average oral language outcomes, but scored above average on measures of executive functioning. These children showed higher scores on both working memory measures and slightly higher scores on selective attention and phonological memory, compared to the DLD-group overall. The third profile was labelled *Mild WM Deficiencies-Average Language and PM* (54.02% of the sample), and was characterized by average scores on oral language, phonological memory, and selective attention measures, but somewhat lower scores (1/2 *SD* below the sample mean) on both working memory measures. The fourth profile was labelled *Strong Development Overall* (13.79% of the sample). Children in this profile scored above average on all measures compared to the overall DLD-sample. Noteworthy, 91.7% of the children in this profile did not have a clinical diagnosis of DLD anymore three years later.

The average latent profile assignment probabilities for individuals assigned to each profile were all above .90, indicating a good clearly separated profile solution (see Table B1). Pairwise profile comparisons per indicator (see Table B2) showed that interference control did not reach a sufficient degree of separation in each pairwise profile comparison. Therefore, it was not considered in describing profile separation. Clear separation on all indicators occurred between the profiles *Weak Development Overall* and *Strong Development Overall* (all values *>* 2.00), and between the profiles *Mild WM Deficiencies-Average Language and PM* and *Strong Development Overall* (all values > 0.85). The *Weak Development Overall* and *Strong EF*-*Average Language and PM* profile were separated on all indicators except sentence repetition. *Weak Development Overall* and *Mild WM Deficiencies*-*Average Language and PM* were separated on all indicators except sentence repetition and visual-spatial working memory. *Strong EF-Average Language and PM* and *Mild WM Deficiencies-Average Language and PM* were separated by the executive functioning indicators, but not by oral language and phonological memory indicators. Lastly, *Strong EF-Average Language and PM* and *Strong Development Overall* were separated by oral language and phonological memory indicators, but not by executive function indicators.

### Nonverbal intelligence

Table 4 summarizes the statistically significant differences in nonverbal IQ scores at time 1 for each pairwise profile comparison. Children in the *Weak Development Overall* profile had an average nonverbal IQ score of 73.79, which is more than 1.7*SD* below the normative mean of 100. Compared to the other three profiles, this profile had the lowest average nonverbal IQ score. Children in the *Mild WM Deficiencies-Average Language and PM* profile had an average nonverbal IQ score of 91.24, which was not significantly different from the average nonverbal IQ score of children in the *Strong Development Overall* profile (i.e., 101.39), but lower than the average nonverbal IQ score obtained by children in the *Strong EF-Average Language and PM* (i.e., 105.05). The nonverbal IQ scores of children in the *Strong EF-Average Language and PM* and *Strong Development Overall* profiles did not differ significantly from each other.

**Table 4.** Wald Chi-square statistic for differences in nonverbal intelligence at time 1 for pairwise comparisons between profiles.

	Weak development overall	Strong EF-average language and PP	Mild WM deficiencies-average language and PP	Strong development overall
Weak Development Overall	–			
Strong EF-Average Language and PP	38.32***	–		
Mild WM Deficiencies-Average Language and PP	23.27***	6.81**	–	
Strong Development Overall	23.04***	.26	3.07	–

Note: Profiles were labelled relative to their position within the overall DLD sample. PP = Phonological Processing, EF = Executive Functioning, WM = Working Memory.

*p < .05, **p < .01, ***p < .001.

### Reading performance: A person-centered approach

**Figure 3.**
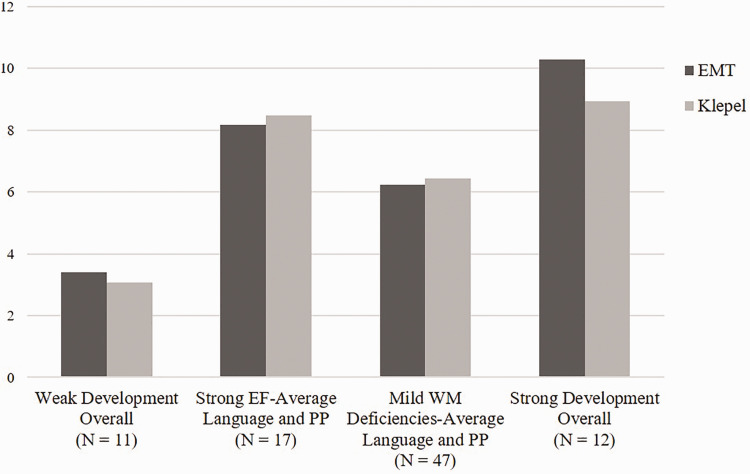
Profile specific mean scores of single-word reading (EMT and Klepel) at time 2. Profiles were labelled relative to their position within the overall DLD sample. PP = Phonological Processing, EF = Executive Functioning, WM = Working Memory.

Figure 3 presents the profile specific age-normed standardized means for single-(non-)word reading at time 2 and Table 5 summarizes the statistically significant differences in single-(non-)word reading at time 2 for each pairwise profile comparison. Children in the *Weak Development Overall* profile scored very weak on single-(non-)word reading at time 2, compared to an age-reference group. Compared to the other three profiles, this was the lowest performing profile on both single-word reading tasks. Children in the *Mild WM Deficiencies-Average Language and PM* profile also achieved weak scores on single-(non-)word reading at time 2. These children scored significantly lower on single-word reading than the children in the *Strong Development Overall* profile, and significantly lower on single-*non*-word reading than the children in the *Strong EF-Average Language and PM*. Children in the *Strong EF-Average Language and PM* and *Strong Development Overall* profiles scored average on single-(non-)word reading.

**Table 5.** Wald Chi-square statistic for differences in EMT (above diagonal) and Klepel (below diagonal) at Time 2 for pairwise comparisons between profiles.

	Weak development overall	Strong EF-average language and PP	Mild WM deficiencies-average language and PP	Strong development overall
Weak Development Overall	–	21.18***	8.52**	28.92***
Strong EF-Average Language and PP	38.16***	–	3.77	2.40
Mild WM Deficiencies-Average Language and PP	13.61***	5.99*	–	11.00***
Strong Development Overall	19.01***	.18	3.70	–

Note: Profiles were labelled relative to their position within the overall DLD sample. PP = Phonological Processing, EF = Executive Functioning, WM = Working Memory.

*p < .05, **p < .01, ***p < .001.

## Discussion and conclusions

This study explored which children diagnosed with DLD early in their lives develop reading problems at later ages by applying a person-centered approach. We examined latent profiles based on measures of oral language proficiency, phonological memory and executive functions, and related these profiles to nonverbal intelligence. The longitudinal design allowed us to explore whether and how the obtained latent profiles at preschool age are related to reading outcomes two years later.

Results showed that the sample had high levels of variability in the severity of DLD and the domains affected by DLD, which is in line with previous studies (Bishop, 2017; Blom & Boerma, 2020; Lancaster & Camarata, 2018). The inclusion of the three broader constructs oral language proficiency, phonological memory, and executive functioning allowed simultaneous consideration of relevant abilities for learning to read (Bishop & Snowling, 2004). The three broader constructs were assembled in profiles in a bottom-up manner, giving rise to four highly separable profiles within the DLD-sample: (1) *Weak Development Overall*, (2) *Strong EF - Average Language and PM*, (3) *Mild WM Deficiencies - Average Language and PM*, (4) *Strong Development Overall.* It is important to note that the labels were chosen based on relative performance within the DLD sample. Below, we first discuss how the different abilities cluster and whether the four profiles relate to nonverbal IQ scores before turning to relations with later reading outcomes.

In each profile, phonological memory patterned with oral language proficiency, supporting that poor phonological memory in preschool children with DLD is accompanied by poor language skills (Snowling et al., 2019). While most profiles show a clustering of the four executive functioning measures (interference control, selective attention, visual-spatial working memory, verbal working memory), the *Mild WM Deficiencies - Average Language and PM* profile suggests dissociation of working memory, on the one hand, and interference control and selective attention, on the other hand. The combination of relatedness and dissociation supports the view that different executive functions share variance, but are also separable (Miyake et al., 2000). Furthermore, it supports findings by Gray and colleagues (2019), showing that working memory profiles are not equivalent to learning disability diagnosis, highlighting the importance of investigating an individuals’ strengths and weaknesses. Note that the specific tasks in our study may have contributed to dissociation patterns: Working memory facilitates performance in selective attention tasks (De Fockert et al., 2001), yet only when selective attention tasks involve a high working memory demand, as for example in selective attention dual-tasks (Spaulding et al., 2008). In the current study, such a task was not used. The *Weak Development Overall* profile showed deficiencies on all measured constructs, but impairments on selective attention and vocabulary stood out. Blom and Boerma (2020), who analyzed data from the same sample as investigated for the purpose of the current study, found that the selective attention scores of children with DLD did not differ from those of TD controls, and vocabulary abilities tend to be relatively (that is, in comparison to grammar) well-developed in DLD (Leonard, 2014). These general patterns do, however, not apply to all children with DLD: The current study shows that there is a subgroup of children with DLD who score low on abilities that are in general spared in DLD, such as selective attention and vocabulary.

The average nonverbal IQ score in the sample was 0.44 standard deviations below the normative mean. This is, however, hardly an accurate reflection of the average IQ scores per profile. The lowest average nonverbal IQ score was found for the *Weak Development Overall* profile, which is 1.7 standard deviations below the normative mean. Children in the *Strong Development Overall* and *Strong EF - Average Language and PM* profile scored around the normative mean. The difference in nonverbal intelligence between the profiles is in line with previous findings, which suggest a relationship between nonverbal intelligence and other predictors of oral and written language (e.g., Rice, 2016; Tomblin & Nippold, 2014). Furthermore, the high variability of nonverbal intelligence supports Bishop’s (2017) recommendation to refrain from the use of the verbal-nonverbal discrepancy as a diagnostic criterium. Surprisingly, the highest nonverbal IQ scores were found for the *Strong EF - Average Language and PM* profile and not for the *Strong Development Overall* profile. In the current study, the Matrices and the Recognition subtests of the Wechsler Nonverbal Scale of Ability (WNV) were used to derive a nonverbal IQ score. The Matrices subtest taps into visual-spatial perceptual reasoning and integration of information, and the Recognition subtest reflects on visual-spatial memory, visual processing and recall (Naglieri & Otero, 2012). These skills are in partly implied in the executive function tasks administered in this study, explaining why children in the *Strong EF - Average Language and PM* obtained relatively high nonverbal IQ scores. The finding that children in the *Strong EF – Average Language and PM* profile have relatively high nonverbal IQ scores support the notion that executive functioning is closely related to nonverbal IQ, yet not identical (Arffa, 2007; Conway et al., 2003; Engelhardt et al., 2016; Kuusisto et al., 2017).

The significant differences between the profiles regarding reading outcomes were in line with our hypothesis. The greatest reading difficulties were found for the *Weak Development Overall* profile followed by the *Mild WM Deficiencies - Average Language and PM* profile. Children with these profiles are at-risk for developing poor reading skills. The *Weak Development Overall* profile had the lowest scores on oral language proficiency, phonological memory, and executive functioning, and it may not be surprising that this group of children is at-risk for reading difficulties. Likewise, it is not unexpected that children in the *Strong Development Overall* profile are *not* at-risk for poor reading outcomes as they appear to possess all the resources necessary for learning to read, according to the extended triangle model. Some studies argue that reading skills of children with DLD-only, *not* diagnosed with comorbid dyslexia, are below age-referenced norms (e.g., Snowling et al., 2019). In our study, children with DLD with the *Strong EF - Average Language and PM* profile or the *Strong Development Overall* profile performed within age-referenced norms on both measures of single word and non-word reading. These results again highlight the importance of acknowledging variability within DLD, and the need for an individual approach with regard to diagnosis and treatment. Furthermore, it reveals that in *research* contexts, comparing predefined groups of children with a pure disorder (e.g., DLD-only) to children with comorbid disorders (i.e., DLD+dyslexia) using arbitrary cut-off scores may not be the best approach to elucidate the relationship between reading difficulties and DLD. Rather, this study shows that using a person-centered approach may be more suited to investigate reading outcomes in the heterogeneous DLD-population.

Perhaps the most intriguing profile is that of the children with the *Mild WM Deficiencies - Average Language and PM* profile, showing that poor reading can also be linked to a more specific constellation of factors. This profile points to the importance of extra-linguistic working memory abilities for single-word reading. The working memory tasks in the current study required processing of verbal and visuospatial information. This profile therefore demonstrates that domain-general aspects of working memory (i.e., the central executive component) are involved in single-word reading, supporting the findings in other research (Gathercole et al., 2005; Smith-Spark & Fisk, 2007; Wang & Gathercole, 2013). Previous studies have revealed that impairments in either oral language proficiency (e.g., Ramus et al., 2013), phonological processing (e.g., Rispens & Baker, 2012), or domain-general executive functions (Booth et al., 2010) are related to reading problems. The results of this study, however, support the hypothesis that children’s reading problems are best represented by a multiple risk or deficiencies model (Bishop & Snowling, 2004). Importantly, the same cognitive impairments in different children with DLD do not necessarily result in the same language and literacy outcomes, because of interactions with other cognitive resources that also vary across children (Bishop & Snowling, 2004). Consequently, combinations of predictors that are assembled using a person-centered approach allow for more meaningful and nuanced conclusions than investigations into the role of single and unique predictors.

## Limitations and future research

A first limitation concerns the sample size. Although the sample size of children with clinically identified DLD is relatively large considering the population, we are aware that this sample size is relatively small for the analytical approach. It is important to note that in LPA statistical power is not only influenced by sample size (Tein et al., 2013). In person-centered approaches, the focus is not to detect statistically significant predictors with sufficient power, but rather to identify the true model based on multiple model fit indices (Solari et al., 2019). Tein and colleagues (2013) argued that distance between the profile indicators (i.e., profile separation) and the number of indicators affected statistical power above and beyond sample size. This study showed that the profiles were highly separated on a general and indicator-specific level. Out of the 48 profile-specific mean comparisons, 31 profile-specific indicators reached separations above 0.85. This study included many indicators, which resulted in higher statistical power (Tein et al., 2013). The sample size limitations were thus to a certain degree reduced by the high degree of separation and the large number of indicators. Larger samples are, however, important to collect more robust knowledge about profiles and to gain more insight into specific profiles, as the current study did not consider profile solutions with small numbers of children with profiles. Therefore, this study is considered an explorative study, which focused on the need for person-centered approaches while investigating individual differences within a heterogeneous population.

A second limitation concerns the measures in the current study. Phonological memory was investigated with a forward digit span task; other phonological abilities, such as phonological awareness, that have also been found to be relevant for word reading, were not tested as part of this study. Executive functioning did include indicators of working memory and interference control, but no measures of mental shifting were available. Interference control did not show to be a powerful indicator for profile formation. This might be due to a large amount of negative flanker effects (18.4%), which implicates a faster response time for the incongruent trials compared to the congruent trials. Negative flanker effects may occur when the response is influenced by perceptual identification instead of response competition, and the flanker task might not only measure interference control but also contrast enhancement (Rouder & King, 2003).

A third limitation concerns the applicability of a person-centered approach for *clinical* purposes. A person-centered approach could identify subgroups that might not exist within the population, as heterogeneity of the population might be better explained by other factors (e.g., a continuum approach) rather than by *clinically* separable subgroups (Lancaster & Camarata, 2018; Williams & Kibowski, 2016). It is, therefore, worth noting that we did not adopt the latent profile approach to detect clinically distinct subgroups, but rather to account for the high levels of variability within the DLD population. The approach enabled us to elucidate the diverse and complex relationship between DLD and reading difficulties, which was the main objective of this study.

## Clinical implications

Although this study mostly provides methodological insights into how to best approach the DLD-population, the findings of our study are of clinical value as it provides a deeper understanding of DLD. Results showed high levels of variability in the severity of DLD and the domains affected by DLD, confirming the heterogeneity of the disorder. Therefore, the results highlight the importance of comprehensively examining an individual's profile, as cognitive impairments in children with DLD can result in different language and literacy outcomes. Knowing an individual’s strengths and needs can contribute to a deeper understanding of factors that underlie language and literacy deficits and lead to the development of more effective reading instructions and interventions by educators and clinicans, tailored to the instructional needs of each individual. Future research is necessary to examine which instructional approaches can be effective for different individual cognitive profiles.

## Conclusions

This study reveals distinct profiles within the DLD-sample based on measures of oral language proficiency, phonological memory and executive functioning. Children with DLD with a weak development on all measures and children with average language and phonological memory with mild working memory deficiencies are most at-risk of developing reading problems. This supports a multiple risk model, in which oral language, phonological memory and executive functions interact and lead to different reading outcomes. The person-centered approach enabled identifying significant relations between reading outcomes and profiles. Therefore, the present study provides a nuanced understanding of the heterogeneity of DLD and its complex relation with reading outcomes. In sum, a person-centered approach seems promising in predicting reading outcomes and does justice to the heterogeneity in DLD.

## Supplemental Material

sj-pdf-1-dli-10.1177_2396941520979857 - Supplemental material for Reading outcomes in children with developmental language disorder: A person-centered approach Click here for additional data file. Supplemental material, sj-pdf-1-dli-10.1177_2396941520979857 for Reading outcomes in children with developmental language disorder: A person-centered approach by Marja C Erisman and Elma Blom in Autism & Developmental Language Impairments
