# Supplementary material for: Reading outcomes in children with developmental language disorder: A person-centered approach
Source: Autism Dev Lang Impair. 2020 Dec 22;5:2396941520979857. doi: 10.1177/2396941520979857 (PMC9620469; doi:10.1177/2396941520979857)
Supplement: sj-pdf-1-dli-10.1177_2396941520979857 - Supplemental material for Reading outcomes in children with developmental language disorder: A person-centered approach [file sj-pdf-1-dli-10.1177_2396941520979857.pdf]

Appendices

Appendix A

Correlations

Table A1

Correlations between Oral Language Proficiency, Phonological Processing, Executive functioning and Reading Ability

Variable	1	2	3	4	5	6	7	8	9	10
1 Vocabulary	-									
2 Morphology	.55**	-								
3 Sentence Repetition	.52**	.61***	-							
4 Phonological Processing	.43***	.44***	.56***	-						
5 Interference Control	.06	.05	.06	.05	-					
6 Selective Attention	-.41***	-.19	-.24*	-.46***	.01	-				
7 Verbal WM	.40***	.35**	.38***	.59***	-.17	-.47***	-			
8 Visual-spatial WM	.41***	.31**	.39***	.47***	-.12	-.44***	.66***	-		
9 Nonverbal Intelligence	.32**	.14	.15	.34**	-.15	-.46***	.45***	.44***	-	
10 Single word reading (time 2)	.16	.08	.16	.38***	-.06	-.33**	.46***	.40***	.43***	-
11 Single non-word reading (time 2)	.14	.09	.24*	.41***	.01	-.30**	.41***	.36**	.34**	.84***

* $p < .05$, ** $p < .01$, *** $p < .001$

Appendix B

Supplemental Model Results LPA

Table B1

Average Latent Profile Probabilities for Most Likely Latent Profile Membership by Latent Profile

	Weak Development Overall	Average Language and PP with Strong EF	Average Language and PP with Mild WM Deficiencies	Strong Development Overall
Weak Development Overall	**0.92**	0.00	0.09	0.00
Average Language and PP with Strong EF	0.00	**0.94**	0.01	0.04
Average Language and PP with Mild WM Deficiencies	0.01	0.04	**0.95**	0.00
Strong Development Overall	0.00	0.06	0.00	**0.94**

Note. Profiles were labelled relative to their position within the overall DLD sample. PP = Phonological Processing, EF = Executive Functioning, WM = Working Memory.

Table B2

Standardized Mean Differences for all Pairwise Profile Comparisons for each Indicator

Variable	*Weak Development Overall* versus *Average Language and PP with Strong EF*	*Weak Development Overall* versus *Average Language and PP with Mild WM Deficiencies*	*Weak Development Overall* versus *Strong Development Overall*	*Average Language and PP with Strong EF* versus *Average Language and PP with Mild WM Deficiencies*	*Average Language and PP with Strong EF* versus *Strong Development Overall*	*Average Language and PP with Mild WM Deficiencies* versus *Strong Development Overall*
Receptive Vocabulary	-1.92	-1.83	-3.43	0.09	-1.51	-1.61
Morphology	-0.96	-0.90	-2.38	0.06	-1.42	-1.48
Sentence Repetition	-0.65	-0.63	-2.72	0.01	-2.07	-2.08
Phonological Processing	-2.86	-2.17	-4.30	0.68	-1.44	-2.13
Interference Control	-0.36	-0.71	-0.25	-0.39	0.12	0.46
Selective Attention	3.20	2.46	3.46	-0.74	0.26	1.00
Verbal WM	-4.07	-1.02	-4.64	3.04	-0.58	-3.62
Visual-spatial WM	-2.47	-0.57	-3.13	1.90	-0.60	-2.56

Note. Profiles were labelled relative to their position within the overall DLD sample. PP = Phonological Processing, EF = Executive Functioning, WM = Working Memory.
